# *Entamoeba histolytica* - tumor necrosis factor: a fatal attraction

**DOI:** 10.15698/mic2015.07.216

**Published:** 2015-07-06

**Authors:** Serge Ankri

**Affiliations:** 1 Department of Molecular Microbiology, Bruce Rappaport Faculty of Medicine, Technion-Israel Institute of Technology, Haifa, Israel.

**Keywords:** Entamoeba, inflammation, TNF, virulence, BspA proteins

*Entamoeba histolytica* is a protozoan parasite, and is the causal agent of human amebiasis whose primary mode of transmission is the ingestion of food and/or water that is contaminated with feces containing *E. histolytica *cysts. Excystation is the stage in a parasite's life cycle, which occurs after the cystic form has been swallowed by the host. When excystation occurs in the intestinal lumen, trophozoites are released and colonize the large intestine, from where the parasite can travel along different life paths which determine the ultimate pathophysiology of amebiasis. *E. histolytica *trophozoites usually reside as a nonpathogenic commensal in the colon of most infected individuals, where they feed on the colon's microbiota 1. In 90% of infected individuals, who are asymptomatic, these trophozoites divide and encyst, and the trophozoites and cysts are subsequently excreted in the feces. However, in the other 10% of infected individuals, symptomatic infection occurs because the trophozoites invade the colonic mucosa by burrowing. The burrows then coalesce to form flask-shaped ulcers and a resultant colitis (amebic dysentery). Disease progression may end with intestinal amebiasis or it may continue in a distal organ as an extraintestinal disease, usually the liver (amebic liver disease). However, rare extraintestinal manifestations of amebiasis with pulmonary, cardiac, and brain involvement can also occur 2.

**Figure 1:**
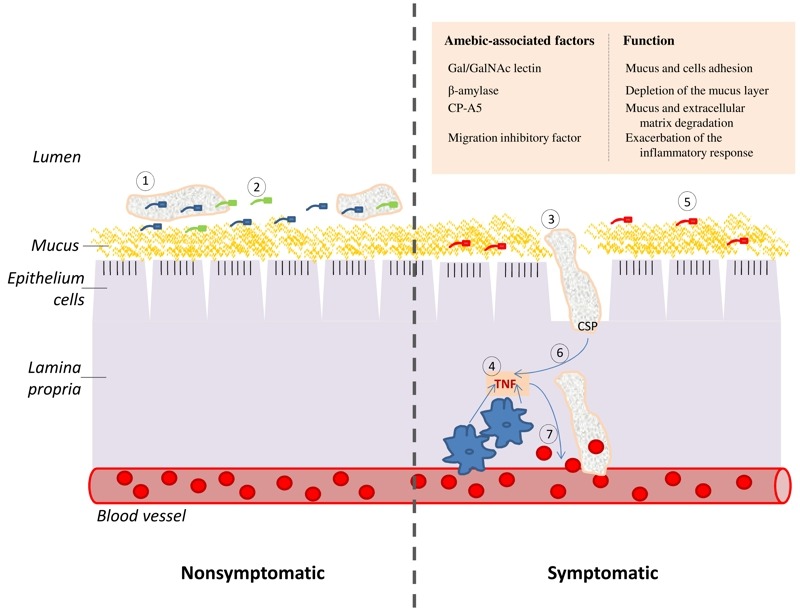
FIGURE 1: TNF and CSP are two essential elements of the cascade of events which lead to amebic colitis. **(Left panel)** Asymptomatic amebiasis: **(1) ** Trophozoites are living as nonpathogenicintestinal commensals, without causing any noticeable damage to the host. **(2) **These trophozoites feed on bacteria and cellular debris that are present in the lumen of the colon and on the surface of the colonic mucus.**(Right panel) **Symptomatic amebiasis: **(3) ** Trophozoites degrade the mucus layer (involvement of β-amylase 4 and CP-A5 8 in this process), trigger a host inflammatory response and induce the collagen remodeling that is required for invasion of the *lamina propria* (involvement of CP-A5) 9. **(4) ** The invasion of the colonic mucosa by *E. histolytica* generates an acute pro-inflammatory reaction which is characterized by the recruitment of macrophages and the production of the pro-inflammatory cytokines, such as TNF. **(5)**Inflammation causes change in the gastrointestinal microbiota 16 and this new flora may be less favorable to the parasite growth. **(6)**The parasite is attracted to the source of TNF production and CSP is essential for this process. **(7)**TNF increase vascular endothelial permeability 18and consequently provides the parasite with an access to erythrocytes. These erythrocytes may represent a better source of nutriment for the parasite than the bacterial flora that is associated with colitis.

One of the first challenges, which *E. histolytica* must overcome in order to cause disease, is its passage through the protective colonic mucus, a complex gel of glycolipids, glycoproteins, and sugar residues, which includes N-acetylglucosamine, N-acetyl-D-galactosamine (GalNAc), D-galactose (Gal), fucose, and sialic acids 3. It has been recently reported that β-amylase is crucial for the depletion of the mucus layer 4. Other parasitic proteins that participate in the colonic invasion of infected individuals are cysteine proteinase (CP), which degrades mucus and the extracellular matrix, and the adherence lectin, Gal/GalNAc, which is crucial for amebic adherence to target cells, to mucus and cytolysis 5 (Fig 1). Regarding the host immune system, it, too, is initially tolerogenic; utilizing both T-regulatory cell activation and secretory immunoglobulin A to suppress inflammatory responses and prevent parasitic contact with the colonic mucus, respectively 1. However, the parasite can be exposed to radically different environments after its invasion of the colonic mucosa. These environments are most notably characterized as being oxygenated, comprised of an extracellular matrix (ECM) (collagen, elastin, laminin, and fibrinogen) 6, and hostile, due to an activated inflammatory immune response. The inflammatory immune response can also be exacerbated by parasite-associated factors, such as the macrophage migration inhibitory factor (MIF) homolog of *E. histolytica *7 and CP-A5 8, which is released by the parasite and activates those human matrix metalloproteinases that are involved in the degradation of the ECM 9.

The triggers, which result in the parasite to no longer be a nonpathogenic commensal and become a pathogen in the colon of an infected individual, have not been clarified. One challenging hypothesis is that this decision is triggered by the host through the secretion of chemoattractants.

An array of such chemoattractants has been previously identified *in vitro* and includes complement component 5a, fibronectin, and unidentified chemoattractants in *E.coli* or erythrocyte extracts 10,11. Another potent chemoattractant is tumor necrosis factor (TNF) 12,13. TNF is one of the cytokines that participates in systemic inflammation and contributes to the acute phase reaction. Although TNF can be synthesized and secreted by many other cell types, such as neutrophils, eosinophils, and mast cells, the main source of TNF is activated macrophages. TNF has a pleiotropic effect on mammalian cells: it can induce apoptosis or assure their survival by activating their proliferation and the nuclear transcription factor NFkB. Usually, TNF contributes to the control of parasitic and bacterial infection like in the case of *Trypanosoma* infection or by triggering an immune response 14. Interestingly, TNF has a cytostatic rather than cytotoxic effect on *E. histolytica* 15. TNF-induced signaling in *E. histolytica* depends on phosphoinositide 3-kinase and Gal/GalNAc and could cause conformational changes in some cytoskeleton-related proteins 13.

In this issue of *Microbial Cell*, Silvestre *et al. *provide the first evidence on the presence of a protein on the surface of the parasite, which senses TNF. This candidate protein which they named CSP belongs to the Bsp-A family of surface proteins. The results of their investigation revealed that CSP is essential for the chemotaxis of *E. histolytica* toward TNF and blocked the invasion of human colon by *E. histolytica*. To uncover this novel finding, Silvestre *et al.* used an antibody against the human TNF receptor (hTNFR) to probe a crude lysate of *E. histolytica* and combined it with a bioinformatics screening of hTNFR homologs in the parasite’s proteome. A s*tructural and bioinformatics analysis *of CSP revealed the presence of an extracellular domain of toll-like receptor 3 (TLR3). Toll-like receptors (TLRs) are non-catalytic receptors usually expressed in macrophages and dendritic cells that recognize structurally conserved molecules in microbes. Importantly, the Toll/interleukin-1 receptor (TIR) domain is present in a typical TLR. The absence of the TIR domain in CSP suggests that CSP corresponds to an ancestral form of the TLR, which is present in higher eukaryotes. To further investigate CSP, they prepared a specific antibody against it and then used this antibody to locate CSP in the parasite by fluorescent confocal microscopy. In control trophozoites, they found that CSP is present on the parasite's surface. When trophozoites were incubated with TNF, the fluorescence signal emanated from intracytoplasmic vesicles of different sizes. Another piece of supporting evidence that CSP is a putative TNF receptor is their finding that CSP was present in the uropod of *E. histolytica* when the parasite was placed on a TNF gradient. They also found that down-regulation of CSP expression in the parasite by antisense RNA impairs TNF chemotaxis and its dispersion within an explant of human large intestine. Collectively, these findings provide convincing evidence that CSP can sense TNF.

The findings of Silvestre *et al.* are important contributions for bettering our understanding of the cross-talk which occurs between *E. histolytica* and the host immune system. Remaining questions are whether CSP is directly involved in the binding to TNF and to other chemoattractants, such as fibronectin. *E. histolytica* activates the inflammatory process by promoting TNF production and CSP is crucial for guiding *E. histolytica* towards TNF-producing cells of the host. One may speculate that this behavior is triggered by its need of nutrients that cannot be satisfied in the inflamed tissues. Inflammation causes change in the gastrointestinal microbiota 16, and these changes may provoke *E. histolytica* to search for alternative sources of nutrients, such as erythrocytes that are often found ingested by trophozoites that were isolated from patients with amebic colitis 17.
